# Risk factors for early postoperative cognitive dysfunction after colorectal surgery

**DOI:** 10.1186/s12871-018-0676-4

**Published:** 2019-01-08

**Authors:** Yuan Zhang, Hong-Guang Bao, Yun-Luo Lv, Yan-Na Si, Liu Han, Hong-Yu Wang, Yu-Jie Gao, Wei-Qing Jiang, Chen Zhang

**Affiliations:** Department of Anesthesiology, Nanjing First Hospital, Nanjing Medical University, 68 Changle Road, Nanjing, 210006 China

**Keywords:** Postoperative cognitive dysfunction, Colorectal surgery, Diabetes, Fasting, SIRS

## Abstract

**Background:**

It has been reported that postoperative cognitive dysfunction (POCD) is correlated with the degeneration of the central nervous system, oxidative stress, inflammation, and endocrine and immune dysfunction. Increased age, predisposed comorbidity, long surgery time, and prolonged stay in the intensive care unit have been reported to be risk factors for developing POCD for cardiac surgery. In the present study, the risk factors of early POCD after colorectal surgery were investigated.

**Methods:**

Eighty patients, who provided informed consents for their participation in this study, were enrolled and received colorectal surgery under general anesthesia. Neuropsychological tests were performed preoperatively and on postoperative day seven. The risk factors for POCD were analyzed using a multivariate logistic regression model.

**Results:**

Nineteen patients were diagnosed with POCD (24.7%). Diabetes history (OR = 8.391 [2.208–31.882], *P* = 0.012), fasting over 3 days after surgery (OR = 5.236 [1.998–13.721], *P* = 0.001) and an SIRS score of > 3 on the second day after surgery (OR = 6.995 [1.948–25.111], *P* = 0.003) were risk factors for early POCD in colorectal cancer patients.

**Conclusion:**

The risk factors for early POCD after colorectal surgery included diabetes history, fasting over 3 days, and an SIRS score of > 3 on the second day.

## Background

Postoperative cognitive dysfunction (POCD) is associated with poor functional recovery, prolonged hospitalization, hospital expenses, need for long-term rehabilitation and mortality [1–6]. Thus, the effective prevention of POCD in elderly patients is very important. One method to prevent the effect of POCD is to identify and avoid its risk factors. A previous study suggested that instable hemodynamics in elderly patients during epidural anesthesia for hip-joint replacement surgery is a risk factor for developing POCD [7]. The dose-dependent effects of intravenous methoxamine infusion during hip-joint replacement surgery decreased the incidence of POCD in a hip-joint replacement surgery population via stabilizing the hemodynamics.

Colorectal cancer (CRC) is one of the most common incident cancers that causes cancer death in China [5]. Its prevalence increases with age, and over 50% of patients with newly diagnosed CRC are older than 70 years of age [8]. Curative resection is recommended for non-metastatic CRC, as well as for metastatic diseases [9]. However, caution should be taken in performing the resection, because the elderly population is vulnerable to postoperative cognitive dysfunction (POCD) [10], and refers to new cognitive impairments after surgery, especially decline in memory and executive functions [11]. However, the risk factors for POCD in CRC patients after surgery remain unclear. Thus, the present prospective study was conducted to reveal the risk factors for POCD in CRC patients.

## Methods

### Patients

The present study was approved by the Ethics Committee of Nanjing First Hospital (ID: KY20131121–01-02) and written informed consent was obtained. A total of 80 Chinese patients, who were 65–75 years old, classified as American Society of Anaesthesiologists (ASA) physical status II or III, and scheduled for elective standard open surgical procedures for CRC from January 2014 to December 2015, were included in the present study. A written informed consent was obtained from each patient or an authorized representative when the patient was unable to provide an informed consent prior to inclusion into the study. Based on the 6th edition of tumor site (T), lymph node invasion (N) and distant metastasis (M), that is, the TNM system [12], the preoperative staging of CRC was performed using whole-body computed tomography (CT) scans and magnetic resonance imaging (MRI). None of these patients received preoperative chemotherapy or radiotherapy.

The participants were required to satisfy the following inclusion criteria: (1) patients with a physical status classification of ASA II or III; (2) patients who were scheduled for elective standard open surgical procedures for CRC under general anesthesia. In addition, the exclusion criteria were as follows: (1) Mini Mental State Examination (MMSE) score of < 23 points; (2) obvious liver or kidney dysfunction; (3) history of mental illnesses or neurological disease; (4) history of taking tranquilizers or antidepressants, or history of alcohol abuse; (5) serious audio-visual obstacles that affects communication; (6) severe vision or hearing loss; (7) recent treatments with sedatives, antidepressants, analgesics, or monoamine oxidase inhibitors; (8) postoperative delirium (a Confusion Assessment Method [CAM] score of > 22 points).

### Standard CRC open surgery and anesthesia

Standard open surgical procedures were performed based on the location, extent and stage of the disease. The anesthesia was induced using 0.03 mg/kg of midazolam, 0.5 μg/kg of sufentanil, 0.3 mg/kg of etomidate, and 0.6 mg/kg of rocuronium. The maintenance of anesthesia was achieved with remifentanil (0.2–0.3 μg/kg/min) and propofol (0.1–0.15 mg/kg/min), and cisatracurium neuromuscular blockade was used when necessary. The Narcotrend® monitor (Narcotrend®-Compact, MT Monitor Technik GmbH & Co. KG, Germany) sensor was placed on the skin of the forehead to record the Narcotrend values during surgery. The Narcotrend index was kept between D1 and E1. During the operation, lactic acid Ringer’s solution and hydroxyethyl starch was intravenously (*i.v.*) infused, and intermittent arterial blood gas analysis was performed. The infusion of red blood cell suspension or frozen fresh plasma was performed when Hb was < 100 g/L or Hct was < 30%. Pain intensity was evaluated daily using the numeric rating scale (NRS), and pain relief was achieved using a patient-controlled analgesia device through the *i.v.* infusion of 150 μg of sufentanil plus 6 mg of tropisetron mesylate, according to the patient’s age, weight and ASA classification.

### Assessment of cognitive function

Delirium was ruled out postoperatively. Cognition was assessed at 1 day before and 7 days after surgery. Neuropsychological assessment was performed in a quiet room, where only the patient and the evaluator were present. All tests were conducted and scored in a standardized manner to minimize possible bias introduced by different evaluators. Project investigators, who were trained in neuropsychological assessment, completed all data scoring and interpretations.

The test battery consisted of MMSE, visual verbal learning test (VVLT), digital span test (DST1), and digital symbol test (DST2), as described in our previous work [13].

In order to rule out the potential bias caused by the learning effect during the cognitive test, 30 subjects were enrolled from the patients relatives (age range: 65–70 years old, body mass index [BMI] range: 20–25, and no gender limitation), and served as healthy controls. These subjects were enrolled based on the above-mentioned inclusion and exclusion criteria, except for the anesthesia or surgery procedure. The same neuropsychological test battery among the healthy controls was repeated twice at each time point, and the difference between the second and first batch of tests was defined as the learning effect. The Z score for each individual was calculated and compared with baseline scores, and divided by the standard deviation (SD) of the learning effect. All neuropsychological functions were tested at 1 day before the surgery and on postoperative day seven. POCD was diagnosed when the Z score was greater than 1.96 or the combined Z score was ≥1.96.

### Statistical analysis and sample size estimation

Variables that potentially correlated to cognitive dysfunction were classified in three categories: preoperative, intraoperative and postoperative factors (Table 1). Continuous data were expressed as mean ± SD. Multivariate logistic regression analysis was conducted to determine the independent predictors for POCD. A *P*-value < 0.05 was considered statistically significant. These statistical analyses were performed using SPSS version 13.0 software (IBM, Inc., Chicago, IL, USA).

**Table 1:** Learning effect and standard deviation (SD) among healthy controls

Item/Instrument	Learning effect	SD
MMSE (0–30)	2.67	0.994
VVLT (0–10)	2.70	1.512
DST1 (0–24)	3.50	1.570
DST2 (0–90)	9.93	3.823

Note: *SD* standard deviation, *MMSE* Mini Mental State Examination, *VVLT* visual verbal learning test, *DST1* digital span test, *DST2* digital symbol test

## Results

The learning effect and SD of healthy controls are presented in Table 1. Among the 77 patients who completed the neurocognitive tests, a total of 19 patients (24.7%) were identified as POCD (POCD group). The test battery scores in the POCD group were significantly lower than those in the non-POCD group (MMSE [*P* = 0.043], VVLT [*P* = 0.004], DST1 [*P* = 0.006], or DST2 [*P* = 0.019]; Table 2).

**Table 2:** Neuropsychological test results

Item/Instrument		non-POCD (*n* = 58)	POCD (*n* = 19)	*P-*value
MMSE (0–30)	Preoperatively	27.10 ± 1.00	27.30 ± 1.80	0.331
	Seven days after surgery	24.20 ± 2.10	23.40 ± 1.10	0.043
VVLT (0–10)	Preoperatively	8.40 ± 1.00	8.70 ± 1.90	0.495
	Seven days after surgery	4.40 ± 2.20	3.20 ± 1.30	0.004
DST 1 (0–24)	Preoperatively	15.80 ± 1.70	16.00 ± 1.90	0.409
	Seven days after surgery	9.30 ± 2.50	7.70 ± 1.90	0.006
DST 2 (0–90)	Preoperatively	23.90 ± 7.40	28.40 ± 7.60	0.103
	Seven days after surgery	13.30 ± 3.60	9.20 ± 2.40	0.019

Note: *POCD* postoperative cognitive dysfunction, *MMSE* Mini Mental State Examination, *VVLT* visual verbal learning test, *DST1* digital span test, *DST2* digital symbol test

Among the potential preoperative risk factors, POCD patients were older (*P* = 0.046) and had higher ASA scores (*P* = 0.009), higher prevalence of diabetes mellitus (*P* = 0.001) and lower education levels (*P* = 0.003), when compared with non-POCD patients (*P* < 0.05). Furthermore, there was no significant difference between the POCD and non-POCD groups in terms of gender (*P* = 0.657), BMI (*P* = 0.057), tumor location (*P* = 0.787) and stage (*P* = 0.099), hypertension (*P* = 0.691), hemoglobin (*P* = 0.221), hematocrit (*P* = 0.346), total protein (*P* = 0.299), and serum albumin (*P* = 0.837).

Furthermore, among the potential operative and post-operative risk factors, POCD patients had a longer fasting time (*P* = 0.000) and higher systemic inflammatory response syndrome (SIRS) score (*P* = 0.000). However, there was no significant difference between the POCD and non-POCD groups in terms of hospital days prior to surgery (*P* = 0.855), operation time (*P* = 0.122), bleeding (*P* = 0.400), transfusion (*P* = 0.845), ΔHb (*P* = 0.601), urine (*P* = 0.244), hypotension (*P* = 0.529), delirium (*P* = 0.420), Intensive Care Unit (ICU) stay (*P* = 0.250), or in-hospital stay (*P* = 0.119). These data are presented in Tables 3 and 4.

**Table 3:** Preoperative characteristics of surgery patients

	non-POCD (*n* = 58)	POCD (*n* = 19)	*P-*value
Age (mean ± SD, years)	69.30 ± 4.60	73.30 ± 4.80	0.046
Gender (male/female)	32/26	10/9	0.657
BMI (mean ± SD)	23.70 ± 2.04	22.30 ± 2.15	0.057
Education level (mean ± SD, years)	7.80 ± 2.60	5.80 ± 2.30	0.003
ASA classification (II/III)	55/3	11/8	0.000
Tumor location			
Proximal colon	15	4	
Distal colon	37	12	0.787
Rectum	6	3	
TNM stage			
Stage I	13	1	
Stage II	23	6	0.099
Stage III	22	12	
Stage IV	0	0	
Hypertension, *n* (%)	18	9	0.691
Diabetes mellitus, *n* (%)	1 (2)	7 (37)	0.001
History of surgery, *n* (%)	9 (16)	7 (37)	0.048
Hb preoperatively (g/L)	129.70 ± 18.40	122.70 ± 21.70	0.221
Hct preoperatively (%)	38.90 ± 4.70	37.40 ± 5.90	0.346
TP preoperatively (g/L)	66.20 ± 5.60	63.70 ± 9.40	0.299
Alb preoperatively (g/L)	38.30 ± 5.60	38.60 ± 5.30	0.837

Note: *Hb* hemoglobin, *Hct* hematocrit, *TP* total protein, *Alb* serum albumin, *SD* standard deviation

**Table 4:** Operative and postoperative variables of surgery patients

	non-POCD (*n* = 58)	POCD (*n* = 19)	*P-*value
Hospital days waiting for surgery (mean ± SD)	2.40 ± 0.49	2.42 ± 0.51	0.855
Operative variables			
Operation time (mean ± SD, minute)	216.02 ± 35.64	225.00 ± 48.42	0.122
Bleeding (ml)	373.28 ± 44.14	381.58 ± 34.20	0.400
Transfusion (ml)	123.28 ± 31.37	202.63 ± 52.05	0.845
ΔHb (%)	9.29 ± 1.51	9.93 ± 1.62	0.601
Urine (ml)	356.90 ± 178.00	310.53 ± 137.00	0.244
Hypotension, *n* (%)	1	1	0.529
Postoperative variables			
Hb 7 days after surgery (g/L)	115.50 ± 15.00	112.80 ± 14.90	0.814
Hct 7 days after surgery (%)	33.80 ± 4.30	33.10 ± 4.10	0.539
TP1 at POD 7 (g/L)	56.80 ± 6.70	56.90 ± 4.80	0.692
Alb1 at POD 7 (g/L)	31.80 ± 3.30	32.50 ± 3.20	0.914
ICU stay, *n* (%)	1 (1.70)	2 (10.50)	0.250
Fasting days (mean ± SD)	2.28 ± 0.62	3.74 ± 0.81	0.000
SIRS score (0–4)	1.88 ± 0.462	3.26 ± 0.991	0.000
Hospital stay (days, mean ± SD)	14.80 ± 3.40	16.30 ± 3.60	0.119

∆Hb = (Hb_0_-Hb_min_)/Hb_0_ × 100%

SIRS, systemic inflammatory response syndrome: (1) temperature > 38 °C or < 36 °C, (2) HR > 90/min, (3) RR > 20/min or PaCO_2_ < 20 mmHg, and (4) WBC > 12 × 10^9^/L or < 4 × 10^9^/L) or immature monocytes > 10%

Hb, hemoglobin

Hct, hematocrit

TP, total protein

Alb, serum albumin

Hypotension, intraoperative blood pressure drops over 20% of that measured at the rest status

POD, postoperative day

SD, standard deviation

ICU, Intensive Care Unit

The multivariate logistic regression analysis revealed the following risk factors for POCD in elderly CRC patients who underwent CRC surgery: diabetes history (OR = 8.391[2.208–31.882]; *P* = 0.012), postoperative fasting duration of ≥3 days (OR = 5.236 [1.998–13.721]; *P* = 0.001), and SIRS score ≥ 3 on the second day after surgery (OR = 6.995 [1.948–25.111]; *P* = 0.003). These data are presented in Table 5.

**Table 5:** Multivariate logistic regression analysis of risk factors for POCD

Variable	Odds ratio	95% confidence interval	*P-*value
Diabetes mellitus	8.391	2.208–31.882	0.012
Fasting time ≥ 3 days	5.236	1.998–13.721	0.001
SIRS score ≥ 3	6.995	1.948–25.111	0.003

Multivariable model adjusted for gender, body mass index, tumor location and stage, hypertension, hemoglobin (Hb), hematocrit, total protein, serum albumin, days in hospital waiting for surgery, operation time, bleeding, transfusion, ΔHb, urine, hypotension, duration of Intensive Care Unit stay, and in-hospital stay

Note: *POCD* postoperative cognitive dysfunction, *SIRS* systemic inflammatory response syndrome

## Discussion

POCD is a common neurological complication in elderly patients who received various surgical procedures, and its incidence is highly dependent on the type of surgery and general anesthesia management [3]. In addition, cognitive dysfunction exists in some CRC patients [14]. Hence, we moved forward and investigated POCD in the patient population that received surgery, in order to determine these relevant risk factors. It was found that the risk factors for POCD in CRC patients included diabetes history, long fasting time after surgery, and high immunoreactive score (IRS) score on the second day after surgery.

In order to increase the strength of the present study, the investigators attempted to standardize the patient background by including only TMN II-III patients who did not receive preoperative chemoradiotherapy, but received the same surgical procedure and peri−/postoperative treatment. However, based on present trial design, the present study was not able to reveal useful findings.

First, history of diabetes is associated with increased risk of developing POCD in CRC patients. The concept of “diabetic encephalopathy” was raised based on the findings that extensive meninges fibrosis, degradation, and the axonal degeneration of neurons were revealed in diabetic brains, suggesting the high chance of neurological impairment in this patient population [6]. Furthermore, diabetes has also been suggested to be an important risk factor of postoperative neurological complications [15]. In the present study, seven (37%) patients with a diabetic history were identified from these 19 POCD patients, when compared with one (2%) patient with diabetic history out of the 58 non-POCD patients. Moreover, the blood glucose levels of all seven diabetic patients were not effectively controlled. These facts support the close relationship between diabetes (or a blood glucose level that is not well-controlled) and POCD in CRC patients.

Second, extended fasting time after surgery is another risk factor for POCD in CRC patients. It has been previously accepted that early eating after surgery increases the incidence of postoperative complications, including abdominal distension, anastomotic fistula, intestinal obstruction, and reverse flow aspiration [16]. However, in recent years, rapid rehabilitation after surgery has increasingly become popular, based on the fact that early oral feeding and exercise after an operation promotes the recovery of bowel functions [17]. Furthermore, early oral feeding within 24 h after rectal surgery significantly reduces postoperative complications, and decreases the risk of anastomotic fistula [18]. In the present study, the standards (2010 edition) for CRC diagnosis and treatment were strictly followed, and liquid diet was prescribed after gastrointestinal decompression to gas pass, while soft oral food was prescribed a week after the surgery. The present study offers evidence that postoperative fasting time over 3 days increased POCD risk in CRC patients.

Third, the inflammatory status after an operation is another risk factor for POCD in CRC patients. The surgery and anesthesia can affect peripheral and central inflammatory factors, and cognitive function. Circulating inflammatory cytokines can facilitate an overreaction in the central inflammatory system, interfere with neuronal activities and synaptic transmission, and ultimately impair the patient’s cognition [19]. Central inflammatory responses are generally represented by SIRS [20], which are caused by the imbalance between inflammation and anti-inflammation, the decrease in immune function, and uncontrollable inflammation [21]. The present study offers evidence that a SIRS score of > 3 in the second day after surgery increases POCD risk in CRC patients.

## Limitations

There were some limitations of the present study. First, the sample size was relatively small due to the inability of patients to perform cognitive tests. Second, pain and opioid treatment are very influential to POCD, and there was a lack of these data in the present study. Third, studies on relevant animal models were not performed to detect the altered protein expression in the hippocampus, which was associated with CRC, in order to support the present hypothesis. Fourth, the present study did not investigate the potential correlation between POCD and body temperature, electrolyte, length of incision, postoperative opioid consumption, or VAS score. Hence, future studies focusing on these issues should be conducted.

## Conclusion

Based on the present observation, POCD occurs in certain CRC patients, and the risk factors for POCD in CRC patients include diabetes history, long fasting time after surgery, and high immunoreactive score (IRS) score on the second day after surgery. These risk factors should be taken into consideration when a CRC surgery is planned.

## Abbreviation

CRC: Colorectal cancer
